# Rh-doped MoTe_2_ Monolayer as a Promising Candidate for Sensing and Scavenging SF_6_ Decomposed Species: a DFT Study

**DOI:** 10.1186/s11671-020-03361-6

**Published:** 2020-06-15

**Authors:** Hongliang Zhu, Hao Cui, Dan He, Ziwen Cui, Xiang Wang

**Affiliations:** 1 Key Laboratory of Biorheological Science and Technology, Ministry of Education, College of Bioengineering, Chongqing University, Chongqing, 400044 China; 2 College of Artificial Intelligence, Southwest University, Chongqing, 400715 China; 3 State Key Laboratory of Power Transmission Equipment & System Security and New Technology, Chongqing University, Chongqing, 400044 China; 4 Chongqing New Oriental School, Chongqing, 400030 China; 5 College of Mobile Telecommunications, Chongqing University of Posts and Telecommunications, Chongqing, 401520 China

**Keywords:** Rh-MoTe_2_ monolayer, First-principles theory, SF_6_ decomposed species, Gas sensor

## Abstract

In this work, the adsorption and sensing behaviors of Rh-doped MoTe_2_ (Rh-MoTe_2_) monolayer upon SO_2_, SOF_2_, and SO_2_F_2_ are investigated using first-principles theory, wherein the Rh doping behavior on the pure MoTe_2_ surface is included as well. Results indicate that T_Mo_ is the preferred Rh doping site with *E*_b_ of − 2.69 eV, and on the Rh-MoTe_2_ surface, SO_2_ and SO_2_F_2_ are identified as chemisorption with *E*_ad_ of − 2.12 and − 1.65 eV, respectively, while SOF_2_ is physically adsorbed with *E*_ad_ of − 0.46 eV. The DOS analysis verifies the adsorption performance and illustrates the electronic behavior of Rh doping on gas adsorption. Band structure and frontier molecular orbital analysis provide the basic sensing mechanism of Rh-MoTe_2_ monolayer as a resistance-type sensor. The recovery behavior supports the potential of Rh-doped surface as a reusable SO_2_ sensor and suggests its exploration as a gas scavenger for removal of SO_2_F_2_ in SF_6_ insulation devices. The dielectric function manifests that Rh-MoTe_2_ monolayer is a promising optical sensor for selective detection of three gases. This work is beneficial to explore Rh-MoTe_2_ monolayer as a sensing material or a gas adsorbent to guarantee the safe operation of SF_6_ insulation devices in an easy and high-efficiency manner.

## Introduction

SF_6_ insulation devices, in high even ultra-high voltage power systems, are one kind of the most important and expensive equipment [1–3], except electrical transformers [4, 5], to guarantee the safe operation of the whole system. These contributions attribute to the strong arc-extinguishing property and high electronegativity of SF_6_ gas that behaves as an insulation media in such devices [6]. However, in a long-running one, SF_6_ could still be decomposed into several low-fluorine sulfides by the power of partial discharge caused by inevitable inner defects of the equipment [7, 8]. Moreover, these by-products will further interact with the surrounding trace water and oxygen, forming some stable chemicals such as SO_2_, SOF_2_, and SO_2_F_2_ and instead deteriorating the insulation behavior of SF_6_ [9]. Therefore, detecting these decomposed species has been regarded as an effective manner to evaluate the decomposing status of SF_6_ and to reflect the operation status of related insulation devices [10].

With the growing attention of transition metal dichalcogenides (TMDs), MoS_2_-based sensors have been proposed for detection of SF_6_ decomposed species [11–13]. These reports have demonstrated the appropriateness and superiority of transition metal (TM)-doped MoS_2_ monolayer for sensing components including SO_2_ and SOF_2_. Besides, a theoretical study on the sensing characteristic of pristine MoTe_2_ monolayer upon SF_6_ decomposed species proves its suitability for sensing SO_2_ [14]. Moreover, recent advancements in chemical vapor deposition (CVD) used for large-scale synthesis of TMDs largely accelerate the development of MoTe_2_ monolayer for gas sensing applications [15–17]. As reported, MoTe_2_ monolayer possesses outstanding carrier mobility, large bond length, and low binding energy, which provides it with high sensitivity upon gas interactions at room temperature [18]. Thus, it is hopeful that MoTe_2_ monolayer is quite a promising candidate for gas sensing, and its application for detection of SF_6_ decomposed species should be further explored.

It is well proved that TM-doped 2D nanomaterials possess stronger adsorption performance and sensing behavior upon gaseous molecules compared with pristine surfaces [19–22]. This is because of the admirable chemical activity and catalytic behavior of TM whose *d* orbital can strongly hybridize with those adsorbed molecules, facilitating the chemisorption and enlarging the charge transfer [23–25]. When it comes to the MoTe_2_ monolayer, to the best of our knowledge there have few theoretical reports on the TM-doping behavior on its monolayer; meanwhile, related adsorption and sensing behaviors of TM-doped MoTe_2_ monolayer upon gases are also less explored. Among the TM elements, rhodium (Rh) with strong catalytic performance has been demonstrated as a desirable TM dopant on other nano-surfaces for gas adsorption [26, 27]. Especially, ref. [26] investigates the Rh doping behavior on the MoSe_2_ monolayer and its enhanced performance for toxic gas adsorption. From this regard, it would be interesting using the first-principles theory to study the Rh doping behavior on the less explored MoTe_2_ monolayer to compare their geometric property and give a better understanding of Rh doping on the TMDs. Beyond that, the adsorption and sensing performances of Rh-doped MoTe_2_ (Rh-MoTe_2_) monolayer upon three SF_6_ decomposed species, namely, SO_2_, SOF_2_, and SO_2_F_2_, were theoretically simulated as well to explore its potential sensing application in some typical areas. The electronic and optical behaviors of Rh-MoTe_2_ monolayer upon gas adsorption provide the basic sensing mechanisms for its exploration as a resistance-type or optical gas sensor to realize the detection of SF_6_ decomposed species. The desorption behavior verifies the potential of Rh-MoTe_2_ monolayer as a gas scavenger to remove these noxious gases in SF_6_ insulation devices, which from another aspect guarantees the safe operation of the power system. This work would be meaningful to propose novel nano-sensing material and its application for evaluating the operation status of SF_6_ insulation devices in an easy and high-efficiency manner.

## Computational Details

All the results were obtained in the Dmol^3^ package [28] based on the first-principles theory. The DFT-D method proposed by Grimme was adopted [29] to better understand the van der Waals force and long-range interactions. Perdew-Burke-Ernzerhof (PBE) function with generalized gradient approximation (GGA) was employed to treat the electron exchange and correlation terms [30]. Double numerical plus polarization (DNP) was employed as the atomic orbital basis set [31]. The Monkhorst-Pack *k*-point mesh of 7 × 7 × 1 was defined for supercell geometry optimizations, while a more accurate *k*-point of 10 × 10 × 1 was selected for electronic structure calculations [32]. The energy tolerance accuracy, maximum force, and displacement were selected as 10^− 5^ Ha, 2 × 10^− 3^ Ha/Å, and 5 × 10^− 3^ Å [33], respectively. For static electronic structure calculations, self-consistent loop energy of 10^− 6^ Ha, the global orbital cut-off radius of 5.0 Å to ensure the accurate results of total energy [34].

A MoTe_2_ monolayer with supercell of 4 × 4 and vacuum region of 15 Å containing 16 Mo and 32 Te atoms was established to perform the whole calculation below. It has been proved that a 4 × 4 supercell is large enough to conduct the gas adsorption process while a 15 Å slab is proper to prevent the interaction between adjacent units [35]. Apart from that, the Hirshfeld method [36] was employed throughout this work to analyze the atomic charge of Rh dopant (*Q*_Rh_) and molecular charge of adsorbed molecules (*Q*_T_). Therefore, a positive value of *Q*_Rh_ (*Q*_T_) represents that the Rh dopant (gas molecule) is an electron donator, while a negative *Q*_Rh_ or *Q*_T_ indicates the related electron-accepting behavior. Only the most favorable configurations of Rh-MoTe_2_ monolayer and adsorption systems are plotted and analyzed in the following parts.

## Results and Discussion

### Analysis of Rh-MoTe_2_ Monolayer

Upon Rh-MoTe_2_ monolayer, four possible adsorption sites are considered, traced as T_H_ (above the center of the hexagonal ring of MoTe_2_), T_Mo_ (at the top of the Mo atom), T_Te_ (at the top of Te atom), and T_B_ (the bridge site between two Te atoms), respectively. The binding energy (*E*_b_) for Rh doping onto the MoTe_2_ monolayer is formulated as:

1 $$ {E}_{\mathrm{b}}={E}_{\mathrm{Rh}\hbox{-} {\mathrm{MoTe}}_2}-{E}_{\mathrm{Rh}}-{E}_{{\mathrm{MoTe}}_2} $$

where${E}_{\mathrm{Rh}\hbox{-} {\mathrm{MoTe}}_2}$,*E*_Rh_, and${E}_{{\mathrm{MoTe}}_2}$represent the energies of the Rh-MoTe_2_ monolayer, Rh atoms, and pristine MoTe_2_ monolayer, respectively.

**Fig. 1:**
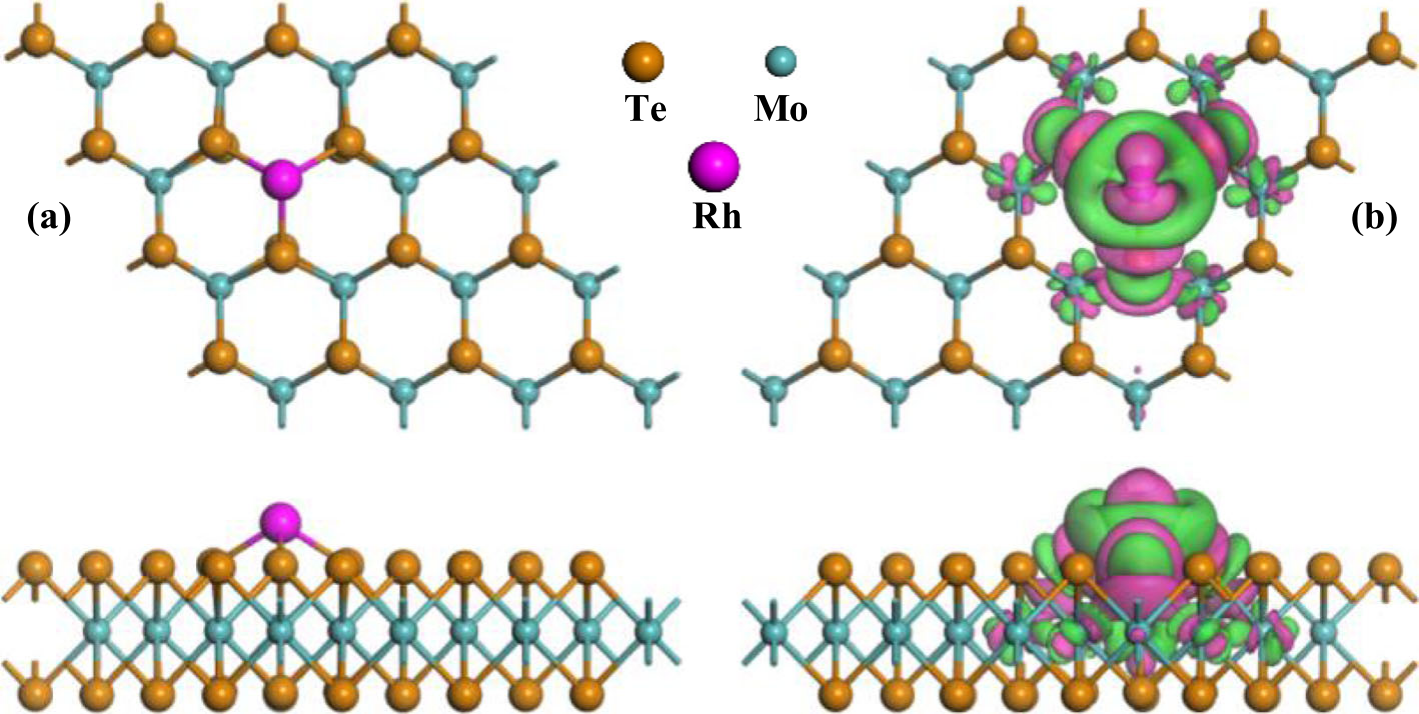
The MSC (**a**) and related EDD (**b**) of Rh-MoTe_2_ monolayer. In EDD, the green (rosy) areas indicate electron accumulation (depletion). The isosurface is 0.005 e/Å^3^

Based on this definition, the most stable configuration (MSC) with the lowest *E*_b_ in line with related electron deformation density (EDD) of Rh-MoTe_2_ monolayer is depicted in Fig. 1. One can see that the Rh dopant is trapped on the T_Mo_ site, forming three covalent bonds with neighboring Te atoms on the upper layer of MoTe_2_ monolayer. Three Rh-Te bonds are measured equally as 2.54 Å, shorter than the sum of covalent radii of Rh and Te atoms (2.61 Å [37]), indicating the formation of chemical bonds for Rh doping on the MoTe_2_ layer. The calculated *E*_b_ of this configuration is − 2.69 eV, much larger than those of − 2.14 eV for T_H_ site, − 1.28 eV for T_Te_ site, and − 2.55 eV for T_B_ site. It is worth noting that the Rh-Te bonds in Rh-MoTe_2_ monolayer are longer than those of Rh-Se bonds in Rh-MoSe_2_ monolayer and the *E*_b_ for Rh doping is smaller on the MoTe_2_ surface in comparison with that of MoSe_2_ counterpart. These indicate the stronger binding force of Rh-Se than Rh-Te bonds. Based on the Hirshfeld method, the Rh dopant behaves as an electron acceptor during doping process, which receives 0.045 e from the MoTe_2_ surface proving its electron-accepting behavior in surface doping [26]. This is in accordance with the EDD in which the Rh atom is mainly surrounded by electron accumulation.

**Fig. 2:**
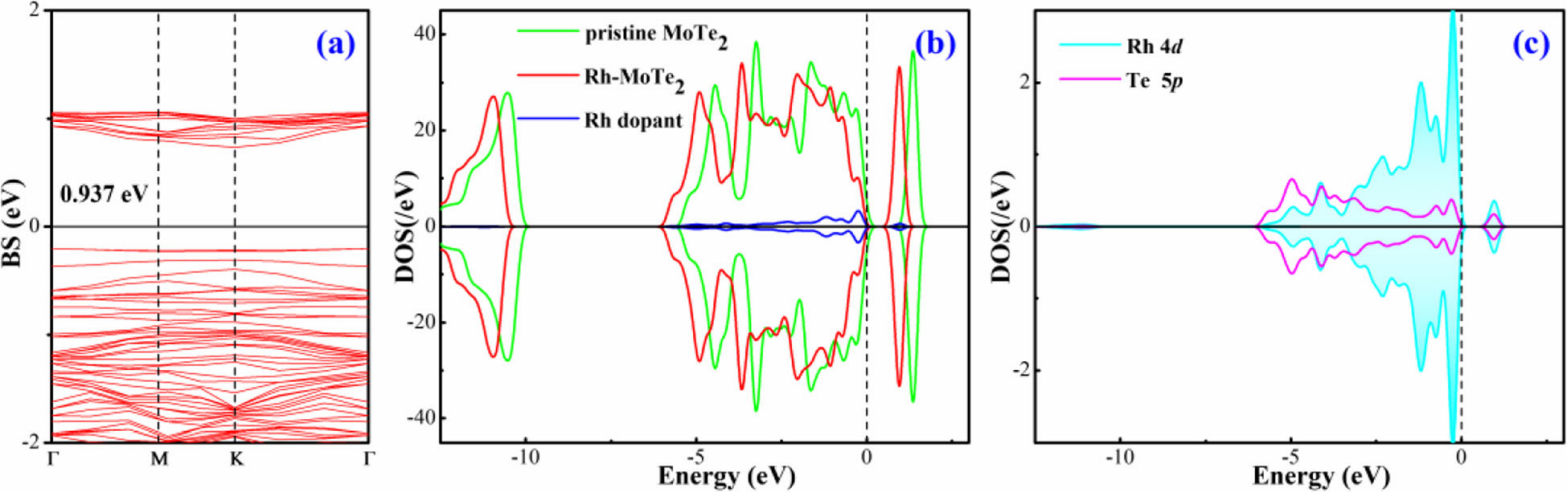
**a** BS of Rh-MoTe_2_ monolayer; **b** DOS comparison between pristine and Rh-doped MoTe_2_ monolayer; and **c** orbital DOS of bonding Rh and Te atoms. The Fermi level is 0

The band structure (BS) and density of state (DOS) of Rh-MoTe_2_ system are depicted in Fig. 2 to better understand the caused change in electronic behavior of MoTe_2_ surface by Rh doping. It is reported that pristine MoTe_2_ monolayer has a direct bandgap of 1.10 eV [38]. In Fig. 2a, the bandgap of Rh-MoTe_2_ monolayer is obtained as 0.937 eV according to the calculations. This indicates that the Rh dopant induces several impurity states within the bandgap of MoTe_2_ system, narrowing the bandgap of the whole system accordingly. Besides, the top of the valence band is localized on the *Г* point and the bottom of the conduction band is on the *K* point, implying the indirect semiconducting property for Rh-MoTe_2_ system. In Fig. 2b, it is seen that the states of Rh dopant contribute largely to the top of the conduction band of pristine MoTe_2_ monolayer and forming several novel DOS peaks around the Fermi level. These peaks seemingly change the electronic behavior of the whole system, reducing its electrical conductivity accordingly. Because the Rh dopant is trapped on the T_Mo_ site forming bonds with Te atoms, the atomic DOS of Rh and Te atoms are plotted to understand the electron hybridization behavior between them. As shown in Fig. 2c, the Rh 4*d* orbital is strongly hybrid with the Te 5*p* orbital from − 5 to 2 eV, accounting for the significant bonding interaction that leads to the formation of chemical bonds of Rh-Te.

### Gas Adsorption Configurations of Rh-MoTe_2_ Monolayer

Based on the relaxed structure of Rh-MoTe_2_ monolayer, the adsorption of SO_2_, SOF_2_, and SO_2_F_2_ molecules onto its surface around the Rh center are fully simulated. Before that, the geometric structures of the three gas molecules should also be optimized as well, as exhibited in Additional file 1: Figure S1. The adsorption energy (*E*_ad_) is used to determine the most stable configuration of each system, formulated as:

2 $$ {E}_{\mathrm{ad}}={E}_{\mathrm{Rh}\hbox{-} {\mathrm{MoTe}}_2/\mathrm{gas}}-{E}_{\mathrm{Rh}\hbox{-} {\mathrm{MoTe}}_2}-{E}_{\mathrm{gas}} $$

in which the ${E}_{\mathrm{Rh}\hbox{-} {\mathrm{MoTe}}_2/\mathrm{gas}}$ and ${E}_{\mathrm{Rh}\hbox{-} {\mathrm{MoTe}}_2}$ are the total energy of Rh-MoTe_2_ monolayer before and after adsorption, whereas *E*_gas_ is the energy of isolated gas molecule. According to this definition, the MSC with the lowest *E*_ad_ could be identified.

**Fig. 3:**
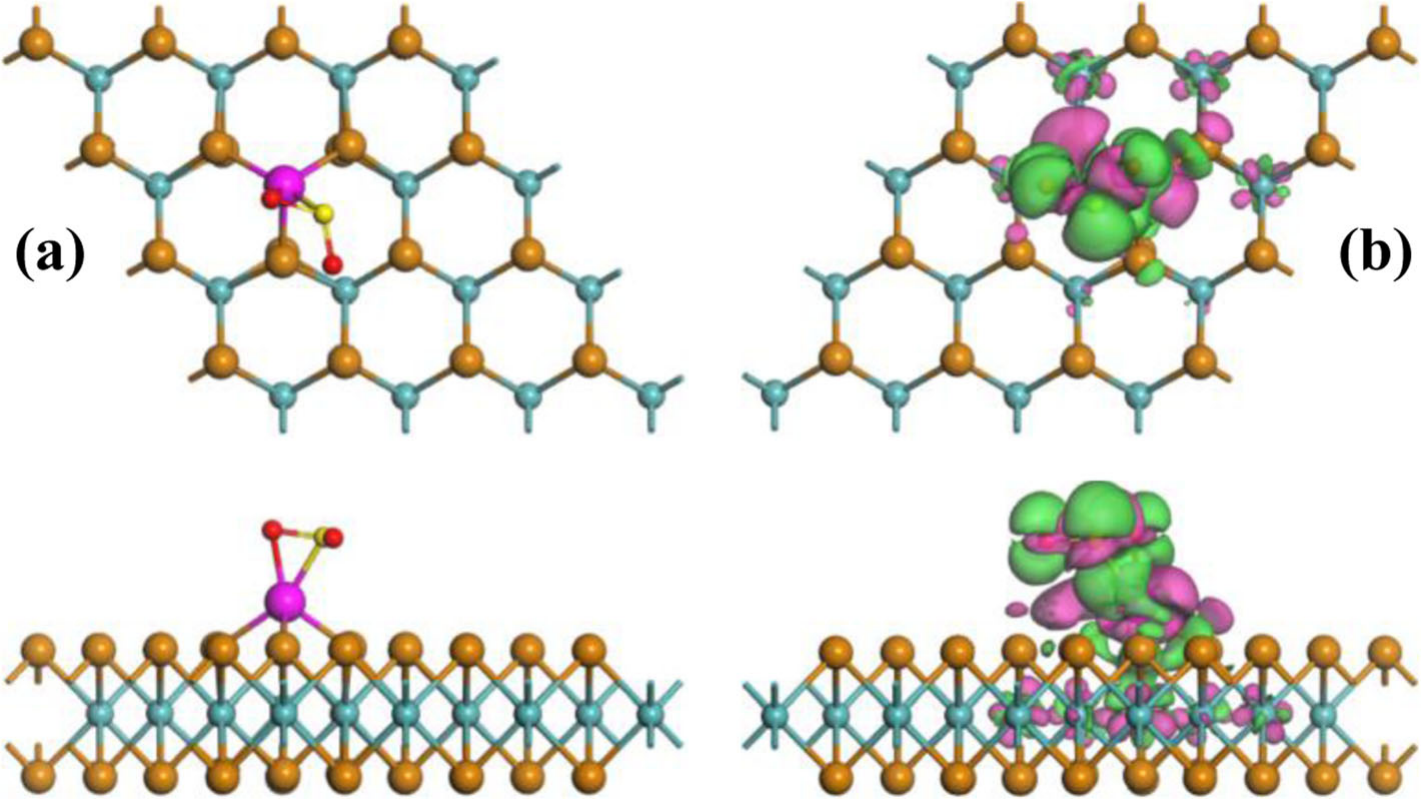
MSC (**a**) and EDD (**b**) of Rh-MoTe_2_/SO_2_ system. In EDD, the green (rosy) areas indicate electron accumulation (depletion), with isosurface as 0.005 e/Å^3^

**Fig. 4:**
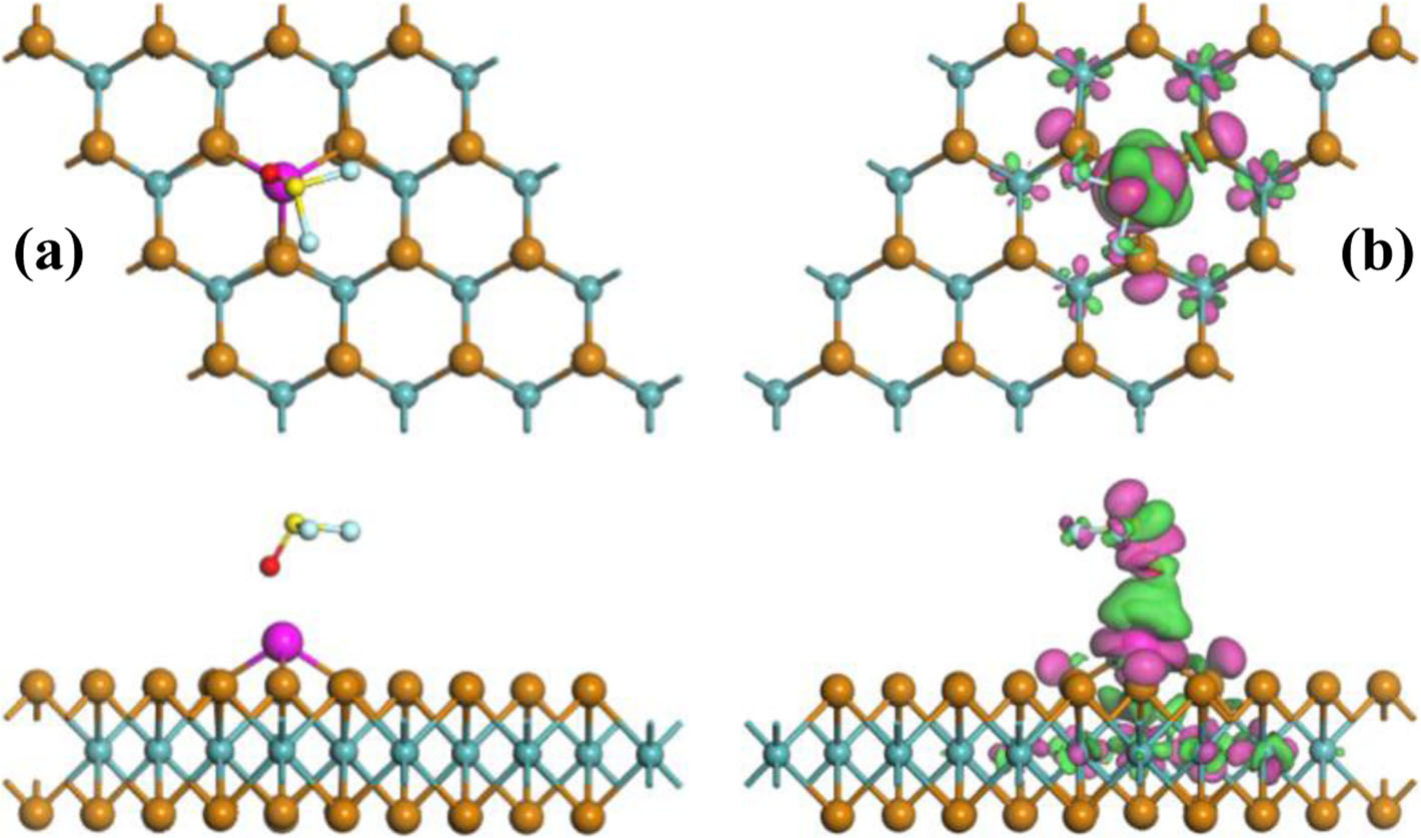
Same as Fig. 3 but for Rh-MoTe_2_/SOF_2_ system

**Fig. 5:**
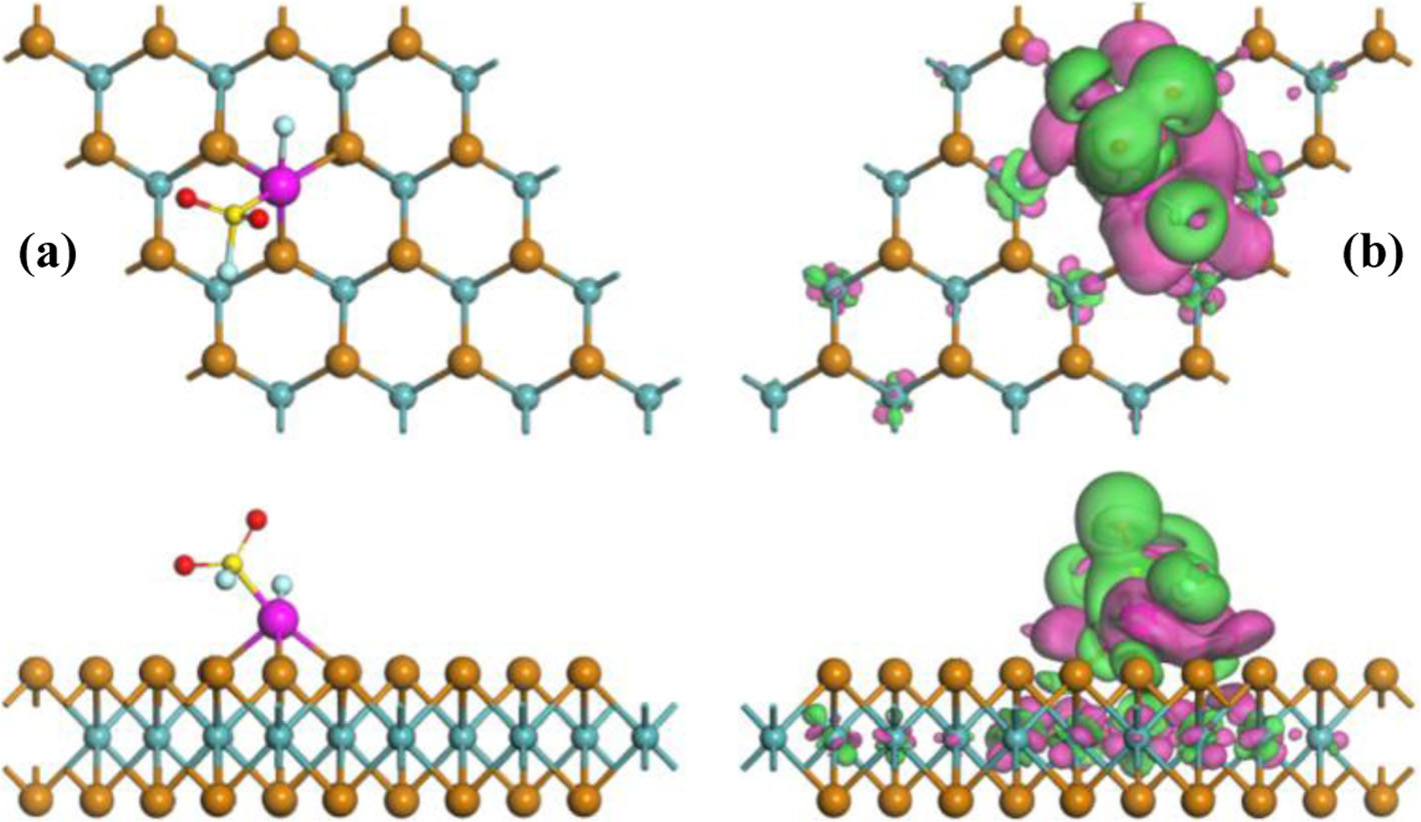
Same as Fig. 3 but for Rh-MoTe_2_/SO_2_F_2_ system

To better understand the charge-transfer behavior during gas adsorption, EDD is also calculated for each configuration. Detailed information for SO_2_, SOF_2_, and SO_2_F_2_ adsorption could be seen in Figs. 3, 4, and 5, respectively. In addition, the adsorption parameters including *E*_ad_, charge-transfer (*Q*_T_), and bond length (*D*) are listed in Table 1.

**Table 1:** Adsorption parameters of Rh-MoTe_2_ monolayer upon SO_2_, SOF_2_, and SO_2_F_2_

Systems	*E*_ad_ (eV)	*Q*_T_ (e)	*D* (Å)
SO_2_ system	− 1.65	− 0.333	Rh-O (2.16); Rh-S (2.36)
SOF_2_ system	− 0.46	0.040	Rh-O (2.25)
SO_2_F_2_ system	− 2.12	− 0.753	Rh-F (2.02); Rh-S (2.26)

For SO_2_ adsorption on the Rh-MoTe_2_ monolayer, one can find from Fig. 3 that the SO_2_ molecule is basically parallel to the MoTe_2_ layer with one O atom and one S atom trapped by the Rh dopant. As listed in Table 1, the newly formed Rh-O and Rh-S bonds are measured as 2.16 and 2.36 Å, respectively, indicating the strong binding force between Rh dopant and SO_2_ molecule. Besides, the *E*_ad_ is obtained as − 1.65 eV implying the chemisorption for the SO_2_ system, and the *Q*_T_ is obtained as − 0.333 e implying the electron-withdrawing behavior of SO_2_. After adsorption, the Rh dopant is negatively charged by 0.017 e, which means it contributes 0.028 e to the adsorbed SO_2_ and the other part of charge (0.305) comes from the MoTe_2_ monolayer. Compared with the adsorption parameters in the MoTe_2_/SO_2_ system (*E*_ad_ = − 0.245 eV; *Q*_T_ = − 0.086 e; *D* = 3.44 Å [14]), one can infer that Rh doping largely enhances the reacting behavior and electronic redistribution for the MoTe_2_ monolayer upon SO_2_ adsorption, making the adsorbent much desirable for gas interaction. Moreover, the S-O bonds in the SO_2_ molecule are separately elongated to 1.50 and 1.58 Å after adsorption, from that uniform 1.48 Å in the gas phase; the three Rh-Te bonds in the Rh-MoTe_2_ monolayer are elongated to 2.58, 2.58, and 2.64 Å, respectively. These deformations imply the geometric activation during adsorption for both nano-adsorbent and gaseous adsorbate, which further confirms the strong chemisorption here. From the EDD distribution, it is found that the SO_2_ molecule is surrounded by electron accumulation, which agrees with the Hirshfeld analysis; and electron accumulation largely surrounds the Rh-S and Rh-O bonds, suggesting the electron hybridization in the formation of new chemical bonds.

In the Rh-MoTe_2_/SOF_2_ system presented in Fig. 4, the SOF_2_ molecule preferred to approach the Rh dopant by the O-end position and the plane made of the S atom and two F atoms are almost parallel to the MoTe_2_ layer. However, there has no obvious evidence suggesting the formation of new bonds between Rh dopant and SOF_2_ molecule. The nearest distance of Rh-O is measured to be 2.25 Å, a little longer than that in the SO_2_ system, and the SOF_2_ molecule does not undergo large geometric deformation after interaction. These findings manifest the relatively weaker adsorption performance of Rh-MoTe_2_ monolayer upon SOF_2_ in comparison with SO_2_. As presented in Table 1, the *E*_ad_ is calculated as − 0.46 eV supporting the physisorption again [39] and the *Q*_T_ is obtained as 0.040 e implying the electron-donating behavior of SOF_2_. According to the EDD, one can see that the electron accumulation is mainly localized on the area between the SOF_2_ molecule and Rh dopant, which implies somewhat hybridization between them, while the electron depletion on the SOF_2_ molecule agrees with the Hirshfeld analysis.

In terms of the SO_2_F_2_ adsorption system, as depicted in Fig. 5, it is found that after optimization, the SO_2_F_2_ molecule tends to be resolved to be a F atom and a SO_2_F group. Both are captured by the Rh dopant forming a Rh-F bond and a Rh-S bond, respectively, with related bond length of 2.02 and 2.26 Å. The newly formed bonds indicate the strong binding force between Rh dopant and SO_2_F_2_ molecule, which combined with the calculated *E*_ad_ of 2.12 eV evidence the chemisorption nature for Rh-doped surface upon SO_2_F_2_ adsorption, similar as that in the SO_2_ system. From the EDD, the electron accumulation is significantly localized on the SO_2_F_2_ molecule, in agreement with the result of *Q*_T_ (− 0.753 e) based on Hirshfeld analysis. On the other hand, a large number of electron depletion is localized on the Rh dopant and a little is on the MoTe_2_ monolayer. In other words, the Rh dopant contributes largely to the charge-transfer to the adsorbed SO_2_F_2_ molecule, manifesting its high electron mobility and strong chemical reactivity [40]. At the same time, the overlap of electron accumulation and electron depletion on the Rh-S and Rh-F bonds suggest the electron hybridization in their formation.

Based on the analysis of adsorption configuration and parameters, one can conclude that the Rh-MoTe_2_ monolayer possesses the best performance upon SO_2_F_2_ molecule, followed by SO_2_ and the last one comes to SOF_2_. In the meanwhile, the Rh dopant can largely affect the electron distribution of this system and therefore dramatically alter the electronic behavior of the Rh-MoTe_2_ monolayer.

### Electronic Behaviors of Rh-MoTe_2_ Monolayer upon Gas Adsorption

**Fig. 6:**
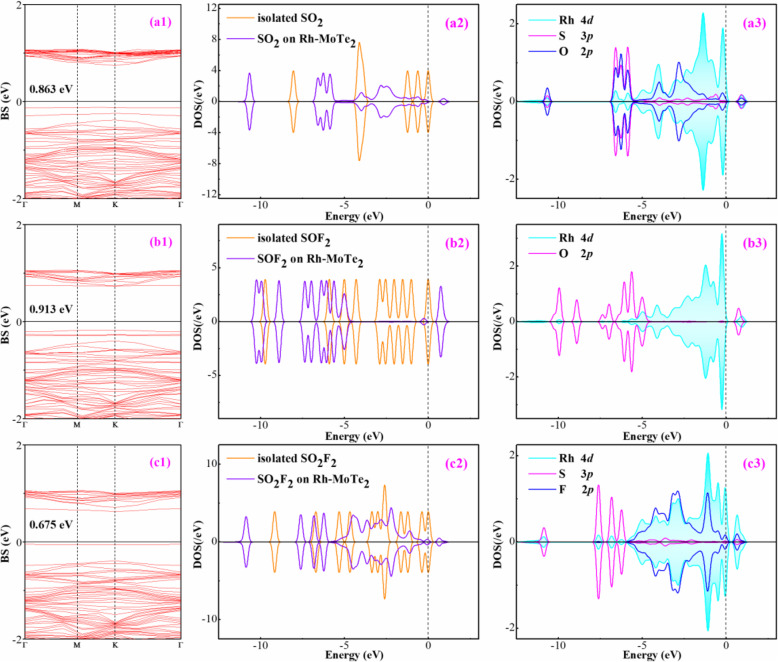
BS and DOS of various systems. (a1)–(a3) SO_2_ system; (b1)–(b3) SOF_2_ system; and (c1)–(c3) SO_2_F_2_ system. In DOS, the dash line is the Fermi level

The band structure (BS) and density of state (DOS) of three adsorption systems are exhibited in Fig. 6 to comprehend the electronic behavior of Rh-MoTe_2_ monolayer in gas adsorption. As above analyzed, Rh-MoTe_2_ monolayer has the best performance upon SO_2_F_2_ adsorption. Thus, from Fig. 6 (c2), it is seen that the molecular DOS of SO_2_F_2_ experiences pronounced deformations, which is integrally left-shifted and some of the states combine to a large one below the Fermi level. From Fig. 6 (c3) where the orbital DOS is shown, it is seen that the Rh 4*d* orbital is highly hybrid with the F 2*p* orbital, and is somewhat hybrid with S 3*p* orbital. From this aspect, it is presumed that Rh-F bond is stronger than that of Rh-S. In the SO_2_ system, the Rh 4*d* orbital is highly hybrid with the O 2*p* orbital and followed by the S 3*p* orbital in Fig. 6 (a3), and one could also presume that Rh dopant has stronger binding force with the O atom rather than the S atom. Due to such hybridization, the molecular DOS of SO_2_ in Fig. 6 (a2) suffers remarkable deformation. As for the SOF_2_ system, one can see in Fig. 6 (b3) that the Rh dopant has little orbital hybridization with the nearest O atom, which supports the weak interaction for SOF_2_ adsorption.

Along with the change of orbital and molecular DOS, the whole state of the gas adsorbed system would be automatically changed compared with the pure Rh-MoTe_2_ system. From Fig. 6 (a1)–(c1) where the BS of the adsorbed system are portrayed, one can see that the BS in the SOF_2_ system does not experience significant deformation in comparison with that of isolated Rh-MoTe_2_ system, while those in the SO_2_ and SO_2_F_2_ system are different, in which some novel states are emerged around the Fermi level, thus narrowing the bandgap largely. Detailedly, the bandgap of the Rh-MoTe_2_ is reduced to 0.863, 0.913, and 0.675 eV after adsorption of SO_2_, SOF_2_, and SO_2_F_2_, respectively. This provides the basic sensing mechanism for Rh-MoTe_2_ monolayer as a possible resistance-type gas sensor.

### Frontier Molecular Orbital Analysis

**Fig. 7:**
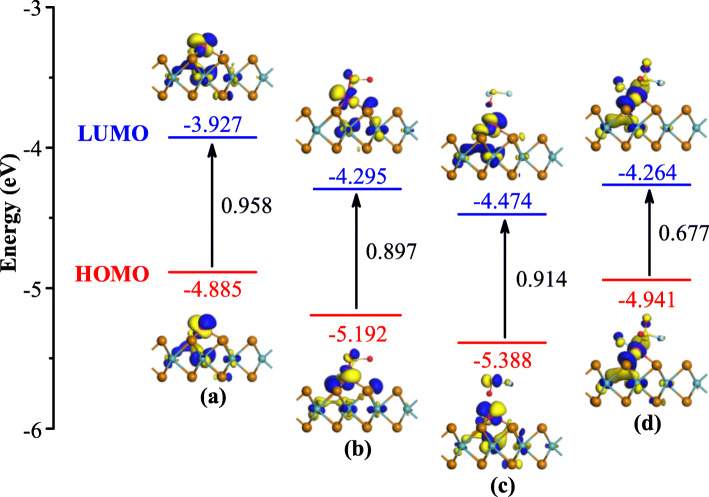
Distributions and energies of FMO in **a** Rh-MoTe_2_ system, **b** SO_2_ system, **c** SOF_2_ system, and **d** SO_2_F_2_ system

To confirm the results based on the BS analysis, the frontier molecular orbital theory is performed to analyze the distribution and energies of frontier molecular orbitals (FMO) of isolated and gas adsorbed Rh-MoTe_2_ surface. The FMO contains highest occupied molecular (HOMO) and lowest unoccupied molecular orbital (LUMO), and the energy gap between them can evaluate the electrical conductivity of the analyzed system [41]. To obtain the accurate results of the energies of FMO, the smearing in this part of calculations is set to 10^− 4^ Å. The distributions and energies of FMO of Ru-MoTe_2_ monolayer before and after gas adsorptions are described in Fig. 7.

From Fig. 7a, one can observe that the HOMO and LUMO are mainly localized at the Rh dopant, suggesting its high reactivity in the surroundings. The energies of HOMO and LUMO are obtained as − 4.885 and − 3.927 eV, respectively, with the calculated bandgap of 0.958 eV. After adsorption of three gas species, as seen in Fig. 7b–d, the FMO distributions of Rh-MoTe_2_ surface are afflicted with different degrees of deformations, where the reaction occurs resulting in the convergence of the electron cloud. Along with these deformations, the energies of FMO have changed accordingly. It is found that the energies of FMO are decreased to different degrees after adsorption of three gases, in which those in SOF_2_ system experience the largest decreases. However, the energy gap in SOF_2_ system undergoes the smallest change in comparison with that of pure Rh-MoTe_2_ system. Specifically, the energy gap of Rh-MoTe_2_ monolayer (0.958 eV) is decreased by 0.044 eV after SOF_2_ adsorption, while is reduced by 0.061 and 0.281 eV after SO_2_ and SO_2_F_2_ adsorption, respectively. These findings indicate that the electrical conductivity of Rh-MoTe_2_ monolayer will decrease after adsorption of three gases and the decrease is the most significant in the SO_2_F_2_ system, which agree with the conclusions from BS analysis. Besides, the energy gaps from frontier molecular orbital theory is basically close to those of bandgaps from BS results, implying the good accuracy of our calculations.

### Sensing Response and Recovery Property

The changes in the bandgap of Rh-MoTe_2_ monolayer after gas adsorption manifest its change in electrical conductivity in related gas atmosphere [42], which can provide the basic sensing mechanism for exploration of Rh-MoTe_2_ monolayer as a resistance-type gas sensor. Besides, the larger change in electrical conductivity would account for a higher sensitivity for gas detection. To identify the possibility of Ru-MoTe_2_ monolayer as a sensor, its conductivity (σ) and sensitivity (*S*) upon three typical gases are calculated using the following formulas [43, 44]:

3 $$ \sigma =\mathrm{A}\cdot {e}^{\left(-{B}_g/2 kT\right)} $$

4 $$ S=\frac{\frac{1}{\sigma_{\mathrm{gas}}}-\frac{1}{\sigma_{\mathrm{pure}}}}{\frac{1}{\sigma_{\mathrm{pure}}}}=\frac{\sigma_{\mathrm{pure}}\hbox{-} {\sigma}_{\mathrm{gas}}}{\sigma_{\mathrm{gas}}} $$

In formula 3, A is a constant, *B*_*g*_ is the bandgap of the analyzed system, *k* is the Boltzmann constant, and *T* is the working temperature. In formula 4, *σ*_gas_ and *σ*_pure_ respectively mean the conductivity of analyzed adsorption system and isolated Rh-MoTe_2_ monolayer. According to such two formulas, it is found that the *S* of certain surface could be obtained just with its bandgap before and after gas adsorption. After calculation, the sensitivities of Rh-MoTe_2_ monolayer upon SO_2_, SOF_2_, and SO_2_F_2_ detection at 298 K are 76.3, 37.3, and 99.4%, respectively. These findings suggest that the Rh-MoTe_2_ monolayer possess the most admirable sensing behavior upon SO_2_F_2_, followed by the SO_2_ and the last one comes to SOF_2_. This order is in accordance with those analysis of adsorption parameter and electronic behavior. Based on these results, it is hopeful that Rh-MoTe_2_ monolayer could realize sensitive detection of SO_2_ and SO_2_F_2_ at room temperature.

The recovery property is also important to evaluate the reusability of the gas sensor, and to reduce the recovery time (τ) of gas desorption from certain surfaces, heating technique is usually considered since the recovery time is related to the temperature (*T*), formulated as [45] $\tau ={A}^{-1}{e}^{\left(-{E}_a/{K}_BT\right)}$. In this formula, *A* is the attempt frequency referring to 10^12^ s^− 1^ [46], *E*_a_ is the potential barrier, determined as equivalent as *E*_ad_ in this work, and *K*_*B*_ is the Boltzmann constant (8.318 × 10^− 3^ kJ/(mol·K)).

**Fig. 8:**
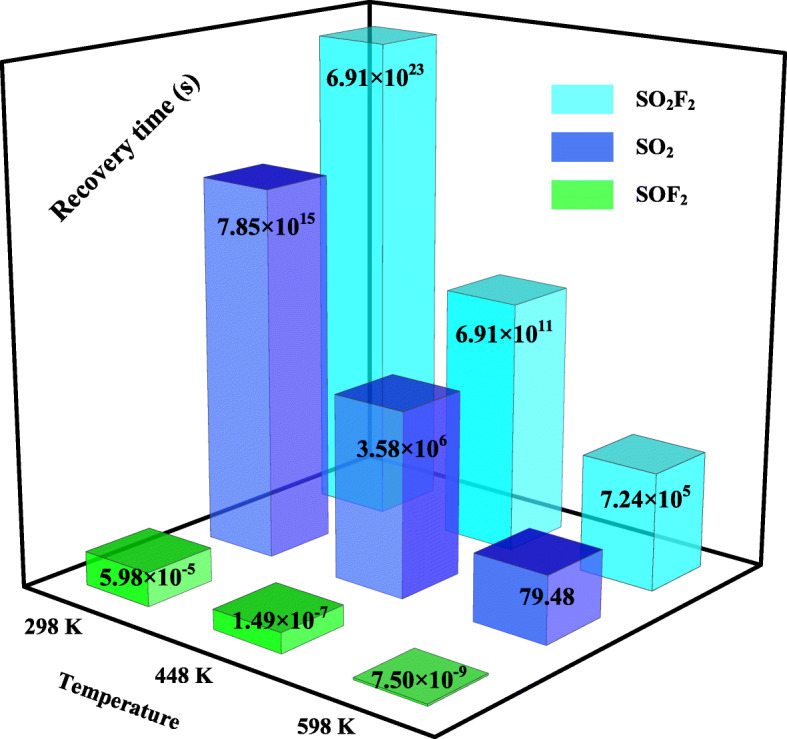
Recovery time of Rh-MoTe_2_ monolayer at various temperatures

Based on the formula, the recovery behavior of Rh-MoTe_2_ monolayer at 298, 448, and 598 K is portrayed in Fig. 8. It can be seen from this figure that the desorption of SO_2_F_2_ and SO_2_ at room temperature are extremely difficult, while for SOF_2_ the recovery time is quite short due to its weak binding force with the Rh-doped surface. Through heating, the recovery time for SO_2_F_2_ or SO_2_ desorption is pronouncedly reduced, and when the temperature increases to 598 K, the recovery time in SO_2_ system (79.48 s) becomes favorable which allow its reusability in several minutes. This supports the potential of Rh-MoTe_2_ monolayer as a reusable gas sensor for detecting SO_2_. On the other hand, the long recovery time for SO_2_F_2_ desorption at 598 K (7.24 × 10^5^) also reflects the strong chemisorption here. Although continuing to increase the temperature can further reduce the recovery time, the thermostability of the sensing material and the high energy consumption in sensing application would be another problem. Given all these, Rh-MoTe_2_ monolayer is not suitable as a sensor for SOF_2_ detection. However, it provides us another thought to propose Rh-MoTe_2_ monolayer as a gas adsorbent to scavenge this noxious gas in SF_6_ insulation devices, thus guaranteeing their safe operation. Moreover, this part of analysis from another aspect reveals the inappropriateness to explore Rh-MoTe_2_ monolayer as a SO_2_F_2_ sensor given the weak interaction with the surface.

### Optical Behavior of Rh-MoTe_2_ Monolayer upon Gas Adsorption

**Fig. 9:**
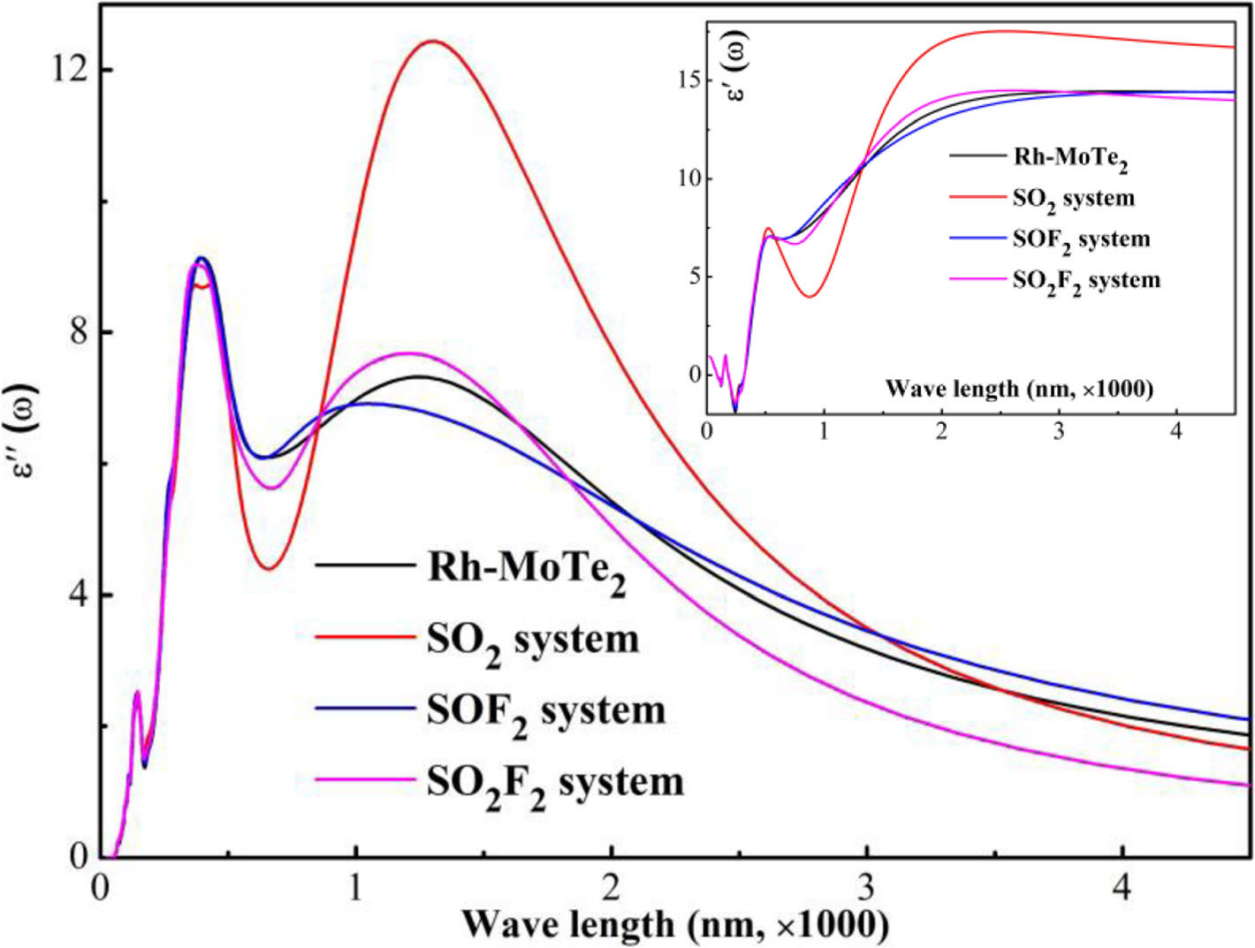
Dielectric function of Rh-MoTe_2_ monolayer

Given the desirable optical property of MoTe_2_ monolayer, the calculation of the dielectric function of Rh-MoTe_2_ monolayer upon gas adsorption is conducted, as displayed in Fig. 9, to illustrate its possibility as an optical gas sensor.

From Fig. 9, it is seen that there have three main adsorption peaks for the isolated Rh-MoTe_2_ monolayer, localizing at 148, 389, and 1242 nm, among which the former two distance are in the range of ultraviolet ray and the last one is in the range of infrared ray. After gas adsorption, the peaks in ultraviolet range suffer small deformation and that in infrared range undergoes significant deformation. Detailedly, the peak intensity at 1242 nm decreases after SOF_2_ adsorption whereas increases after SO_2_ and SO_2_F_2_ adsorption, and the blue shift could also be identified in the SOF_2_ system. Therefore, it could be assumed that Rh-MoTe_2_ monolayer is a promising optical sensor for sensitive and selective detection of three gases by infrared device.

In short, it is worth adding that this work makes a progressive research for proposing novel nanomaterials to realize the detection of SF_6_ decomposed species through various techniques, which would be significant to fulfil the evaluation of SF_6_ insulation devices in an easy and high-efficiency manner.

## Conclusions

In this paper, the potential application of Rh-MoTe_2_ monolayer as a gas sensor for detection of SF_6_ decomposed species is explored, which mainly contains two aspects: (1) Rh doping behavior on the intrinsic MoTe_2_ monolayer and (2) adsorption and sensing behaviors of Rh-MoTe_2_ monolayer upon SO_2_, SOF_2_, and SO_2_F_2_. It is found that the Rh dopant prefers to be doped on the MoTe_2_ surface through the T_Mo_ site with *E*_b_ of − 2.69 eV, exerting great electron hybridization with the Te atoms. The adsorption performance of Rh-MoTe_2_ monolayer upon three gases are in order as SO_2_F_2_ > SO_2_ > SOF_2_, in which chemisorption is identified in SO_2_F_2_ and SO_2_ systems while physisorption in SOF_2_ system, as further supported by the DOS analysis. Rh-MoTe_2_ monolayer is a promising resistance-type gas sensor for recycle detection of SO_2_ with a response of 76.3%, is a desirable adsorbent for SO_2_F_2_ removal from the SF_6_ insulation device, and is promising as an optical sensor for selective detection of three gases. All in all, Rh-MoTe_2_ monolayer is a potential sensing material for detection of SF_6_ decomposed species. This work is meaningful to propose novel nano-sensing material and to realize the effective evaluation of SF_6_ insulation devices in an easy and high-efficiency manner.

## Methods Section

This work means to explore novel 2D sensing materials using first-principle theory for application in electrical engineering, through detecting the SF_6_ decomposed species to evaluate the operation status of high-voltage insulation devices.

## Supplementary information

**Additional file 1: Figure S1**. Geometries of (a) SO_2_, (b) SOF_2_, and (c) SO_2_F_2_. The black values are bond length while the orange values are bond angles.

## Data Availability

The data at present cannot be shared because they are still in study in our following research.

## Abbreviations

TMDs: Transition metal dichalcogenides
TM: Transition metal
CVD: Chemical vapor deposition
PBE: Perdew-Burke-Ernzerhof
GGA: Generalized gradient approximation
DNP: Double numerical plus polarization
*Q*_Rh_: Atomic charge of Rh dopant
*Q*_T_: Molecular charge of adsorbed molecules
*E*_b_: Binding energy
MSC: Most stable configuration
EDD: Electron deformation density
BS: Band structure
DOS: Density of state
*E*_ad_: Adsorption energy
*D*: Bond length
FMO: Frontier molecular orbitals
HOMO: Highest occupied molecular
LUMO: Lowest unoccupied molecular orbital
