# Supplementary material for: Rh-doped MoTe2 Monolayer as a Promising Candidate for Sensing and Scavenging SF6 Decomposed Species: a DFT Study
Source: Nanoscale Res Lett. 2020 Jun 15;15:129. doi: 10.1186/s11671-020-03361-6 (PMC7295872; doi:10.1186/s11671-020-03361-6)
Supplement: Supplementary file 1 — Additional file 1: Figure S1. Geometries of (a) SO2, (b) SOF2, and (c) SO2F2. The black values are bond length while the orange values are bond angles. [file 11671_2020_3361_MOESM1_ESM.docx]

*Fig. S1 Geometries of (a) SO_2_, (b) SOF_2_ and (c) SO_2_F_2_. The black values are bond length while the orange values are bond angles.*
